# Severe Gastric Mucosal Necrosis Due to Giant Paraesophageal Hernia

**DOI:** 10.7759/cureus.24564

**Published:** 2022-04-28

**Authors:** Patrick Duplan, Humdoon Choudhry, Mohammad Memon, Daniel Klein, Dilip Ghanekar

**Affiliations:** 1 Internal Medicine, HCA Florida Bayonet Point Hospital, Hudson, USA; 2 Medicine, HCA Florida Bayonet Point Hospital, Hudson, USA; 3 Gastroenterology, HCA Florida Bayonet Point Hospital, Hudson, USA

**Keywords:** hiatal hernia, dysphagia, erosive ulcers, paraesophageal hiatal hernia, gastric mucosal necrosis, gastric volvulus

## Abstract

Hiatal hernias occur when part of the intra-abdominal contents protrude into the chest cavity. Paraesophageal hernia (PEH) is a type of hiatal hernia that is chronic and usually asymptomatic. Although patients may not present with alarming symptoms, the complications of PEH may be severe if left untreated. Hiatal hernias can be further categorized based on the degree of herniation. The most common subtype is a type I hiatal hernia, which occurs when the gastroesophageal junction (GEJ) herniates into the chest cavity. Type II, III, and IV PEH are when the GEJ, a portion of the stomach, and abdominal viscera herniate into the thorax. A PEH is usually chronic and asymptomatic. However, patients may present with vomiting, dysphagia, bloating, and abdominal pain. Complications of PEH may include gastric mucosal necrosis, perforation, strangulation, erosive ulcers, and gastric volvulus. This report discusses a case of a 71-year-old male patient who had multiple complications arising from a large PEH that required emergent treatment due to its nebulous presentation.

## Introduction

Hiatal hernias may present with various complications primarily affecting those who are of 50-years of age and older. There are four types of hiatal hernias, and each is classified according to its degree of herniation. Type I occurs when the gastroesophageal junction (GEJ) herniates into the chest cavity and is the most common type of hiatal hernia. The remaining types (II, III, and IV) are paraesophageal hernias (PEH) which occur when the GEJ, a portion of the stomach, and abdominal viscera herniate into the thorax. A PEH is usually chronic and asymptomatic, however, patients may present with vomiting, dysphagia, bloating, and abdominal pain. 

While PEHs are often asymptomatic, they may lead to severe complications if incarceration occurs [1]. Complications of PEH may include gastric mucosal necrosis, perforation, strangulation, erosive ulcers, and gastric volvulus. A PEH can be partially or completely herniated. Partial herniations typically manifest with only mild symptoms, whereas complete herniations are often acute and may lead to obstruction. Approximately 1% of complete herniation cases require emergent surgery [2]. A literature review using the PubMed database of severe complications of PEH, including gastric necrosis and perforation, reported only five cases over the last 20 years [3,4]. 

The clinical presentation of PEH depends on multiple factors. These factors include but are not limited to the speed of onset, degree of obstruction, and type of gastric volvulus. High-grade PEH can cause acute gastric volvulus, which can further be complicated by Borchardt's triad. Borchardt's triad consists of unproductive retching, epigastric pain, and the inability to pass a nasogastric tube as described in the case below. This triad is observed in nearly 70% of patients with PEH [5]. A PEH can also present with other nonspecific symptoms such as nausea, vomiting, hiccups, and hematemesis. Chronic symptoms such as postprandial pain, dysphagia, breathlessness, and vomiting have also been reported [6]. In rare situations, severe retching can lead to mucosal tear and ischemia, which eventually present with clinical signs of hemorrhage [7]. Routine surveillance and complete gastroenterology workup are therefore warranted to avoid long-term complications. The following case report describes a patient with severe gastric mucosal necrosis due to a giant PEH.

## Case presentation

A 71-year-old male with a past medical history of hypertension, hyperlipidemia, dyspepsia, chronic alcohol use, tobacco use, and moderate paraesophageal hernia presented with epigastric abdominal pain to our hospital. The patient noted the pain had been ongoing for one week and was associated with a two-day history of constipation and dark stools. Epigastric pain was described as deep, achy in nature, and 6/10 intensity. The patient had associated intermittent retching with no nausea. The patient’s last colonoscopy was done 21 years before presentation and was unremarkable for any pathology. 

In the emergency department, vital signs were unremarkable except for blood pressure of 220/130 mmHg. Physical exam was significant only for an obese abdomen and tenderness to light palpation in the epigastric region. His initial laboratory studies were notable for: white blood cell of 10.6 uL (8.5-10.1), hemoglobin of 19 g/dl (13.7-17.5), blood urea nitrogen of 21 ng/dl (7-18), creatinine of 1.40 ng/dl (0.60-1.30), glomerular filtration rate of 49.96 mL/min/1.73m squared, total bilirubin of 1.10 mg/dl (0.20-1.00), pro-B-natriuretic peptide of 798 pg/ml (0-125), lipase 422 unit/L (73-393), troponin I high sensitivity of 484 ng/L (<78), and gastric occult blood positive. 

**Figure 1:**
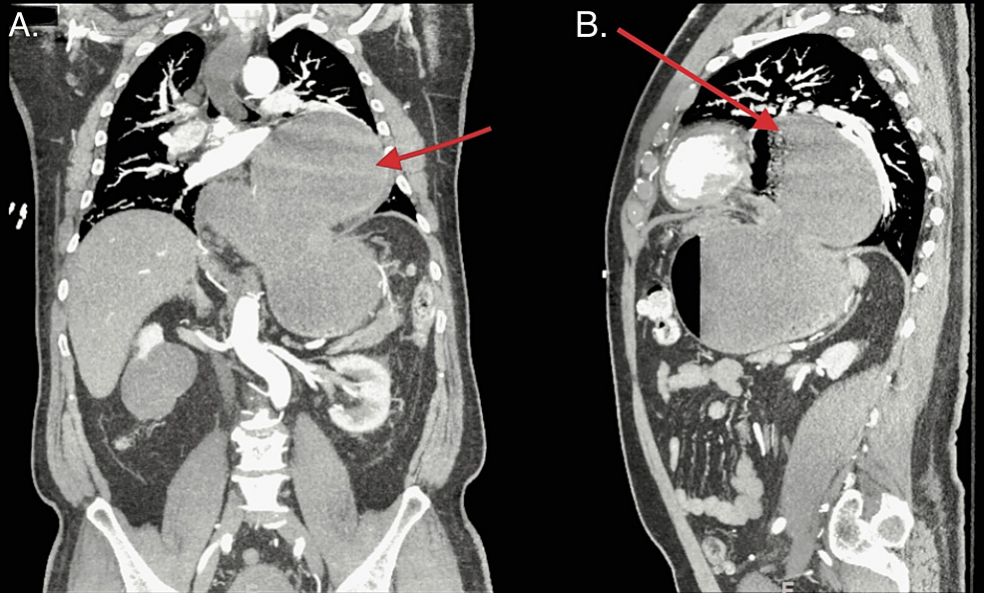
CT of the chest and abdomen demonstrating a large hiatal hernia (red arrow) and a dilated, fluid-filled esophagus.

**Figure 2:**
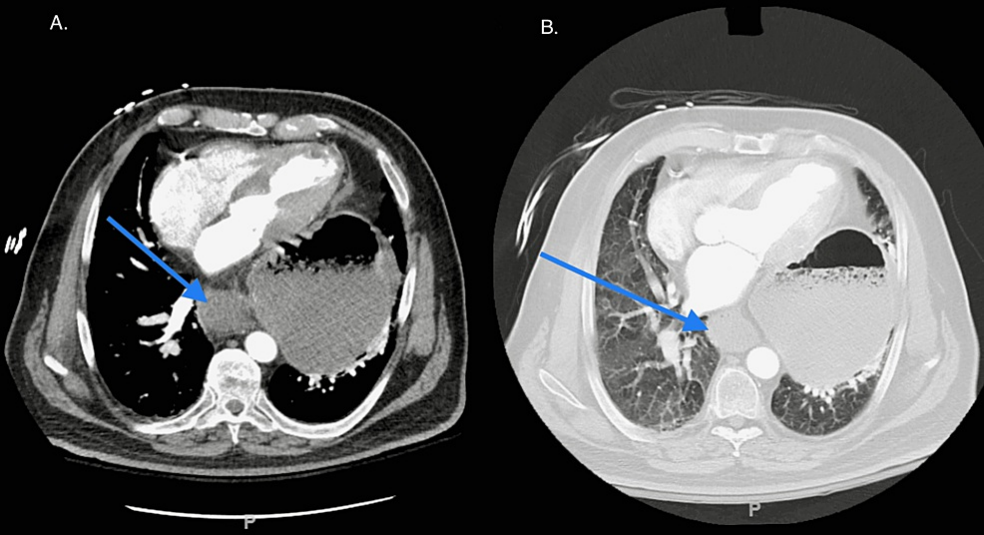
Transverse section of CT chest demonstrating a large hiatal hernia and a dilated, fluid-filled esophagus (blue arrows).

Initial chest X-ray revealed a hiatal hernia which was larger when compared to an x-ray from 8 months prior. A CT of the abdomen with contrast revealed a large hiatal hernia and a dilated, fluid-filled esophagus (see Figures 1, 2). A CT angiography of the chest was negative for any other acute pathology.

**Figure 3:**
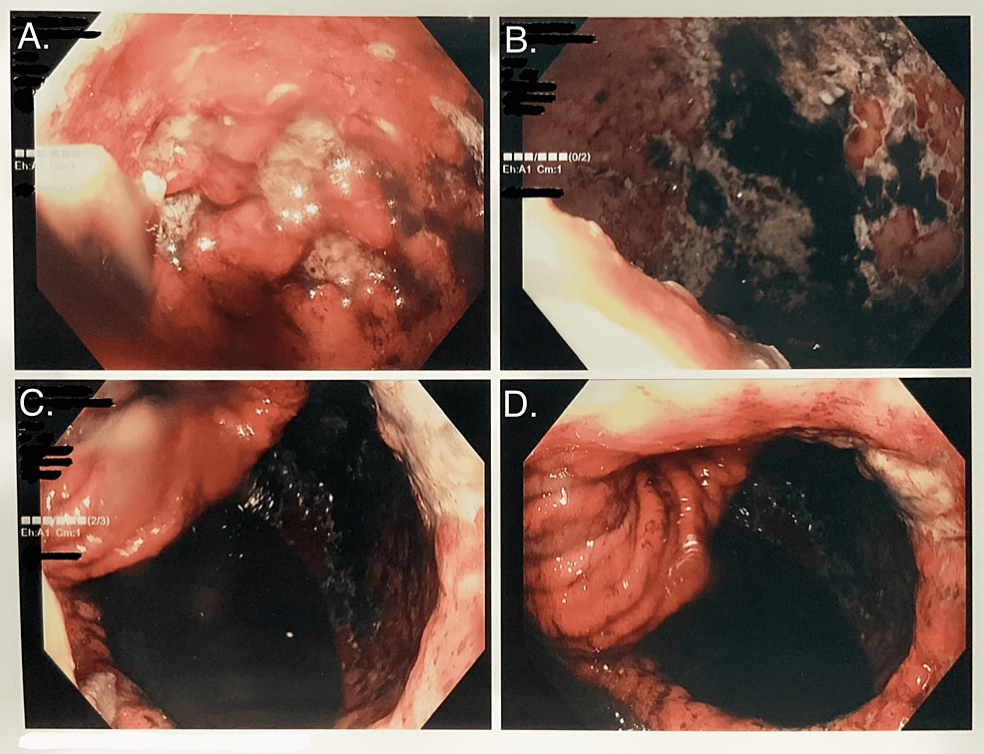
EGD showing severe ulcerative esophagitis and gastritis, gastric mucosal necrosis, and very large paraesophageal hiatal hernia.

**Figure 4:**
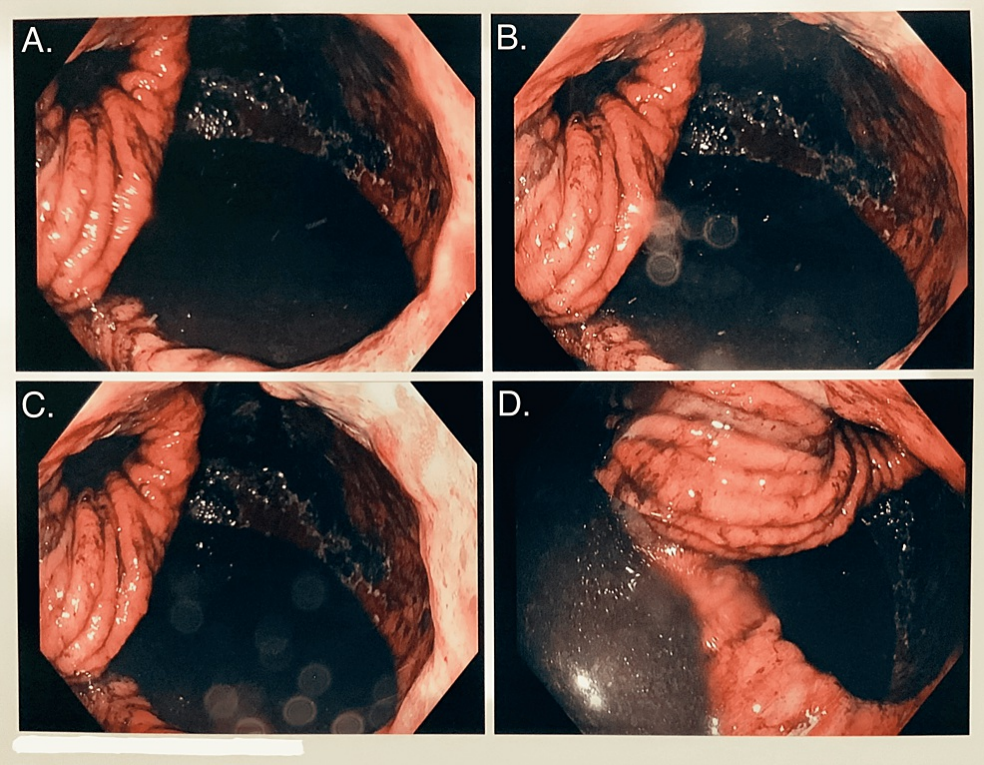
EGD showing a closer look at severe ulcerative esophagitis and gastritis, gastric mucosal necrosis, and very large paraesophageal hiatal hernia.

The patient was admitted for endoscopic evaluation of the hernia. Esophagogastroduodenoscopy (EGD) was remarkable for severe ulcerative esophagitis, severe gastritis with evidence of gastric mucosal necrosis, and high-grade PEH, which precluded advancement of the scope to the antrum (see Figures 3, 4). During the EGD, the patient was suctioned, which yielded 1.3 L of coffee-ground fluid. Subsequently, the patient's intravenous pantoprazole dose was increased to 40 mg twice daily. General surgery was consulted at the recommendation of the gastroenterologist. 

Furthermore, the patient presented with elevated high-sensitivity troponins and was evaluated by cardiology to rule out acute coronary syndrome. The patient was subsequently taken for a diagnostic left heart catheterization (LHC) by interventional cardiology. The LHC was remarkable for severe atherosclerotic coronary artery disease, dominant right coronary artery with 95% stenosis of ostia and proximal disease, mid-segment of the left anterior descending artery with tandem focal lesions of 95% and 90%, normal left ventricular systolic function, and normal left ventricular end-diastolic pressure.

Per cardiology recommendations, cardiothoracic surgery was consulted to evaluate for a possible coronary artery bypass graft (CABG) procedure. The patient’s underlying PEH was later deemed as high-grade with volvulus by general surgery who recommended transfer for PEH repair and fundoplication. Given the complexity of this case, the patient was transferred to a nearby tertiary care center for high-risk CABG, hiatal hernia repair, and fundoplication. 

## Discussion

A PEH can be diagnosed on plain films by the presence of gas-filled viscus in the upper abdomen or lower chest [8]. The proposed pathogenesis of gastric necrosis is likely from gastric outlet obstruction or ischemic insults to the vascular pathways. Vascular impairment coupled with gastric reflux contributes to direct mucosal injury, thus creating necrotic tissues [9]. The upper esophagus is more vascularized than the distal third, which receives blood supply from the inferior phrenic and left gastric arteries. Although the distal esophagus is relatively hypovascular, the presence of a complex vascular network within the submucosa of the esophagus renders esophageal and gastric necrosis a rare occurrence [10].

In the case discussed above, both esophageal and gastric ischemia were likely multifactorial but incited by the presence of PEH and complicated by torsion. Other studies have demonstrated a similar presentation, coupled with other symptoms such as nausea and nonspecific abdominal pain [11]. Coincidentally, the patient had underlying risk factors such as hyperlipidemia, alcohol abuse, and tobacco abuse which further increased the risk of mucosal necrosis. Few studies have identified risk factors, including hypertension, malignancy, and diabetes, which further aggravate gastric and esophageal necrosis. Such risk factors collectively lead to hypoperfusion, which weakens the gastric lining, makes stomach mucosa vulnerable to direct damage from acidic gastric content, and increases the risk for gastric necrosis [10]. Therefore, we deduced that PEH combined with the patient’s risk factors caused accelerated damage to the patient’s gastric mucosa. This led to severe ulcerative esophagitis and gastritis similar to nonsteroidal anti-inflammatory drugs (NSAID)-induced gastritis [12,13].

Conservative treatment modalities for PEH include restoring flow and reducing reflux with intravenous fluids, minimal oral intake, total parenteral nutrition, and proton pump inhibitors [14]. Patients with PEH can be effectively managed by supportive care that helps to halt further damage and necrosis before definitive surgery. High mortality rates are strongly linked to type III or IV PEH in older patients with other comorbidities, which increases the importance of initiating treatment and preventing rapid deterioration [15].

Multiple techniques should be a part of the diagnostic framework to evaluate PEH, which can also cause acute or chronic gastric volvulus (GV). Such framework should include gastrointestinal studies such as an EGD and thoracoabdominal CT. In chronic GV, EGD is typically performed preoperatively to evaluate for the presence of gastric malignancy. In acute GV, EGD can help identify the source of an active bleed, mucosal congestion or ulceration. Our patient in this case study showed severe ulcerative esophagitis, gastritis, and multiple Cameron ulcers. Cameron ulcers are seen in 5% of PEH patients. The pathogenesis of Cameron ulcers is not clear, however, it has been hypothesized that these ulcers may arise from mechanical trauma secondary to high-grade PEH [16]. High-grade PEH can cause failure to pass the scope through the pylorus, as observed in this case. If perforation is suspected, proper care and precaution should be taken [17].

A thoracoabdominal CT is a sensitive technique used to confirm the diagnosis of GV. It can help reveal evidence of necrosis, locate the anatomical defects, identify pneumatosis in the stomach wall, and exclude other medical conditions [18]. Once the anatomy of the PEH is identified, prompt treatment should be initiated. 

A surgical approach is recommended for symptomatic patients, especially those presenting with obstructive symptoms and volvulus [19]. In patients with severe gastric necrosis secondary to PEH, surgical intervention is essential to resolve symptoms and reduce the risk of incarceration or volvulus. The primary aim of surgery is to relocate herniated structures to their original position in the abdomen, repair the hiatal opening with a mesh, and perform gastropexy or fundoplication to prevent recurrence [20].

## Conclusions

Our report discusses a rare case of PEH complications, etiology, diagnostics, treatment, and management. While the majority of PEH cases are chronic and asymptomatic, severe complications may arise from large PEH that require emergent treatment. We hope this case report improves the understanding of PEH and its severe gastrointestinal complications and further enhances the medical literature pertaining to this subject.
